# l-Isoleucine Administration Alleviates DSS-Induced Colitis by Regulating TLR4/MyD88/NF-κB Pathway in Rats

**DOI:** 10.3389/fimmu.2021.817583

**Published:** 2022-01-11

**Authors:** Xiangbing Mao, Rui Sun, Qingxiang Wang, Daiwen Chen, Bing Yu, Jun He, Jie Yu, Junqiu Luo, Yuheng Luo, Hui Yan, Jianping Wang, Huifen Wang, Quyuan Wang

**Affiliations:** ^1^ Institute of Animal Nutrition, Sichuan Agricultural University, Chengdu, China; ^2^ Key Laboratory for Animal Disease-Resistance Nutrition of China Ministry of Education, Chengdu, China; ^3^ Key Laboratory of Animal Disease-Resistant Nutrition and Feed of China Ministry of Agriculture and Rural Affairs, Chengdu, China; ^4^ Key Laboratory of Animal Disease-Resistant Nutrition of Sichuan Province, Chengdu, China

**Keywords:** isoleucine, inflammation, DSS-induced colitis, TLR4/MyD88/NF-κB pathway, rats

## Abstract

Inflammatory bowel disease (namely, colitis) severely impairs human health. Isoleucine is reported to regulate immune function (such as the production of immunoreactive substances). The aim of this study was to investigate whether l-isoleucine administration might alleviate dextran sulfate sodium (DSS)-induced colitis in rats. In the *in vitro* trial, IEC-18 cells were treated by 4 mmol/L l-isoleucine for 12 h, which relieved the decrease of cell viability that was induced by TNF-α (10 ng/ml) challenge for 24 h (*P <*0.05). Then, in the *in vivo* experiment, a total of 44 Wistar rats were allotted into 2 groups that were fed l-isoleucine-supplemented diet and control diet for 35 d. From 15 to 35 d, half of the rats in the 2 groups drank the 4% DSS-adding water. Average daily gain, average daily feed intake and feed conversion of rats were impaired by DSS challenge (*P <*0.05). Drinking the DSS-supplementing water also increased disease activity index (DAI) and serum urea nitrogen level (*P <*0.05), shortened colonic length (*P <*0.05), impaired colonic enterocyte apoptosis, cell cycle, and the *ZO-1* mRNA expression (*P <*0.05), increased the ratio of CD11c-, CD64-, and CD169-positive cells in colon (*P <*0.05), and induced extensive ulcer, infiltration of inflammatory cells, and collagenous fiber hyperplasia in colon. However, dietary l-isoleucine supplementation attenuated the negative effect of DSS challenge on growth performance (*P <*0.05), DAI (*P <*0.05), colonic length and enterocyte apoptosis (*P <*0.05), and dysfunction of colonic histology, and downregulated the ratio of CD11c-, CD64-, and CD169-positive cells, pro-inflammation cytokines and the mRNA expression of *TLR4*, *MyD88*, and *NF-κB* in the colon of rats (*P <*0.05). These results suggest that supplementing l-isoleucine in diet improved the DSS-induced growth stunting and colonic damage in rats, which could be associated with the downregulation of inflammation *via* regulating TLR4/MyD88/NF-κB pathway in colon.

## Introduction

Gut functions and integrity play a vital role for health and growth in humans and animals (1). Inflammatory bowel disease (IBD), especially colitis, is a kind of chronic non-specific disease, which is derived from intestinal epithelium erosion and inflammatory invasion. However, with recurrent and persistent features, IBD impairs the life quality of patients (2). Researchers have already obtained some IBD models, namely, cell models (i.e., tumor necrosis factor (TNF)-α induction), and rodent models [i.e., oral gavage of dextran sulfate sodium (DSS)] (3, 4). Recent studies have illustrated that some nutritional ways (such as essential oils, probiotics) may improve the inflammation in IBD models (5–7).

Isoleucine, known as one of branched chain amino acids, is important for some physiological functions of humans and animals (8–11). Through reviewing the previous studies, it is found that isoleucine is important to maintain immunity in the *in vivo* and *in vitro* trials, which is involved into the regulation of immune organs, immune cells, and immunoreactive substances (such as immunoglobulins, cytokines, and host defense peptides) (12). Our recent study also showed that l-isoleucine administration could relieve rotavirus infection *via* affecting immune response, namely, the mRNA expression and concentration of inflammation-related cytokines in the ileal mucosa of weaned piglets (13). Thus, l-isoleucine administration could regulate the inflammatory response, and be used to control and cure DSS-induced colitis. However, there were no related studies.

Toll-like receptor 4/myeloid differentiation primary response gene 88/nuclear factor-kappa B (TLR4/MyD88/NF-κB) pathway would be stimulated in IBD models, which could further increase the generation of pro-inflammatory cytokines in colon (14, 15). In our recent study, l-isoleucine administration could regulate the mRNA expression of TLR3, NF-κB, and inflammatory cytokines in the ileal mucosa of rotavirus-infected piglets (13). Thus, the aim of this study was to verify the hypothesis that dietary l-isoleucine supplementation might alleviate gut damage and inflammation in the rats with DSS-induced colitis, and preliminarily analyze the possible mechanism in this process.

## Materials and Methods

### Cell Culture Experiment

#### Cell Culture

The IEC-18 cell line (rat ileal epithelial cells) was purchased from iCell Bioscience Inc. (Shanghai, China). IEC-18 cells were cultured with DMEM medium (Gibco Laboratories Life Technologies Inc., Grand Island, NY) with 10% fetal bovine serum (Gibco Laboratories Life Technologies Inc., Grand Island, NY), 0.1 U/ml insulin (iCell Bioscience Inc., Shanghai, China), and 1% antibiotics (Penicillin-Streptomycin Solution; Gibco Laboratories Life Technologies Inc., Grand Island, NY) at 37°C in 5% CO_2_.

### Cell Viability Assay

The viability of IEC-18 cells was measured with the Cell Counting Kit-8 (CCK8; Beyotime, Jiangmen, China) according to the manufacturer’s instructions. In brief, IEC-18 cells were seeded in 96-well plates at 1.0 × 10^4^ cells/well. Following 20 h culture, the varying concentrations (0, 2, 4, 8, and 16 mmol/L) of l-isoleucine or the varying concentrations (0, 5, 10, and 20 ng/ml) of TNF-α were added to the cells (n = 10 or 12). At 12 and/or 24 h after l-isoleucine and TNF-α treatment, CCK8 solution was added and incubated for 2 h. Cell viability was determined with BioTek Synergy HT microplate reader (BioTek Instruments, Winooski, VT) at an absorbance of 450 nm. Through these, the suitable treating-dose and -time of l-isoleucine or TNF-α were obtained.

Then, we measured whether l-isoleucine treatment could influence the effect of TNF-α on cell viability. Briefly, IEC-18 cells were seeded in 96-well plates at 1.0 × 10^4^ cells/well. Following 20 h culture, 0 or suitable dose of l-isoleucine were supplemented to the cells for suitable treating-time (n = 16). The media were removed. Cells were washed three times with PBS and incubated with free-serum and free-antibiotics media containing 0 or suitable dose of TNF-α for 24 h (n = 8). CCK8 solution was added and incubated for 2 h. Cell viability was measured with BioTek Synergy HT microplate reader (BioTek Instruments, Winooski, VT) at an absorbance of 450 nm.

### Animal Experiment

#### Animals, Diets, and Experimental Design

The trial protocol was approved by the Animal Care Advisory Committee of Sichuan Agricultural University. All operations were carried out at the Experimental Farm of Sichuan Agricultural University with Regulations on Animal Welfare and Animal Testing. A total of 44 male Wistar rats weaned at 21 d were from Chengdu Dashuo Experimental Animal Co., Ltd. (Chengdu, China). All rats were individually housed in a temperature-controlled room (21–23°C) on a 12L:12D photoperiod, and had food and water *ad libitum*.

A non-purified rodent diet based on maize, soybean meal, wheat flour, and fishmeal was purchased from Chengdu Dashuo Experimental Animal Co., Ltd. (Chengdu, China), which met the Chinese National Standard (GB14924.3-2010). Either 1.00% (w/w) l-isoleucine or 0.68% (w/w) l-alanine (isonitrogenous control) was supplemented to the non-purified rodent diet. Feed mixture was executed by the Chengdu Dashuo Experimental Animal Co., Ltd. (Chengdu, China). In this non-purified rodent diet, the nutritional levels were crude protein (20%, w/w), Ca (1.36%, w/w), and total P (1.02%, w/w).

After 3 d of acclimatization, according to body weight, 44 Wistar rats were allotted into 2 groups: one fed with the l-isoleucine-supplemented diet or one fed with the l-alanine-supplemented (isonitrogenous control) diet (n = 22) for 35 d. From 15 to 35 d, half of the rats in the 2 groups drank the ultrapure water with 4.0% (w/v) DSS (40,000 mol wt; Herbon International Co., Ltd., Heyuan, China) while the others drank the ultrapure water. *Via* measuring feed intake in each day and body weight per 3 days, average daily gain (ADG), average daily feed intake (ADFI), and feed conversion (F/G) of all rats were calculated.

### Evaluation of Colitis

The colitis of rats was evaluated as described previously (16). Briefly, body weight per 3 days, daily hemoccult or presence of gross blood, and daily stool consistency were measured in all rats, and then the disease activity index (DAI) was determined by an investigator blinded to the protocol by scoring changes in weight, hemoccult positivity or gross bleeding, and stool consistency. The method of scoring is shown in **Supplemental Table 1**.

### Sample Collection

On the 36th day, after weighing, all rats were anesthetized with sodium pentobarbital, and the abdomen was exposed. Blood of all rats were collected from abdominal aorta. Serum samples were made by centrifuging blood at 3,500×*g* for 10 min, and stored at −20°C. Then, the colons of all the rats were quickly isolated, and its length was measured. Four rats were randomly chosen in each group, and segments of colon (about 5 cm) were collected, placed into ice-cold PBS solution, and used to determine cell cycle and apoptosis. In the other rats, segments of colon (about 1 cm) were fixed in 4% paraformaldehyde. The residual colons of all the rats were immediately frozen in liquid nitrogen and stored at −80°C until measurement.

### The Analysis of Serum Urea Nitrogen and Free Amino Acids

Serum urea nitrogen level was measured with an assay kit from the Nanjing Jiancheng Biochemistry Institute (Nanjing, China) according to the manufacturer’s instructions. The concentrations of serum free amino acids were analyzed by ion-exchange chromatography with physiological fluid analysis conditions (L-8900 AA Analyzer, Hitachi, Japan) as described previously.

### Colonic Enterocyte Apoptosis Assay

Colonic epithelial cells were immediately separated from colons of each rats, and ground to form the cell suspension that was filtered through a 300-mesh nylon screen. After washing cells twice with ice-cold PBS, cells were suspended in PBS at 1 × 10^6^ cells/ml. Then, 100 µl cell suspension were transferred into 5 ml culture tubes, and 5 µl Annexin V-FITC (Invitrogen Life Technologies, Inc., Carlsbad, CA) and 10 µl propidium iodide (PI; Invitrogen Life Technologies, Inc., Carlsbad, CA) were added. The cell suspension was gently vortexed and incubated at room temperature for 15 min in the dark. Lastly, 400 µl of Annexin V binding buffer (Invitrogen Life Technologies, Inc., Carlsbad, CA) was added in each tube, and the apoptotic cells were determined with CytoFlex flow cytometer (Beckman Coulter, Inc., Brea, CA).

### Colonic Enterocyte Cell Cycle Assay

After cell suspension was made in colon, 1 ml cell suspension was transferred into 5 ml culture tubes, and 1 ml PBS was added. The cell suspension was centrifuged at 300×*g* for 5 min. Cells were re-suspended with 2 ml ice-cold ethanol (70%), and incubated at 4°C for 15 min. Following centrifuging at 300×*g* for 5 min, the supernatant was abandoned, and 400 µl PBS was added. Then, 50 µl PI (BD Pharmingen, San Jose, CA) and 50 µl RNase (BD Pharmingen, San Jose, CA) were added into cell suspension, and the cell suspension was incubated at 4°C for 30 min. Finally, cell cycle distributions were analyzed with CytoFlex flow cytometer (Beckman Coulter, Inc., Brea, CA).

### Histopathology of Colon

In histopathological assay, colon segments fixed in 4% paraformaldehyde were embedded in paraffin. The consecutive sections (5 µm) were stained by using Masson’s trichrome (MT) and hematoxylin–eosin (H&E). Then, the histopathological changes in colon were observed at 40× magnification with an Olympus CK 40 microscope.

### Colonic Immunohistochemistry Analysis

After being fixed in 4% paraformaldehyde, colon segments were embedded in paraffin. The sections (2 µm) were deparaffinized and hydrated, and pre-treated with 3% H_2_O_2_ at room temperature for 15 min, and then heated in 10 mmol/L citrate buffer (pH 6.0) for 3 min. After washing with PBST three times, the sections were blocked with 10% goat serum at room temperature for 60 min. Then, the sections were incubated overnight at 4°C with 1:200 dilution of rabbit anti-CD11c antibody (Biorbyt, Cambridge, UK), anti-CD64 antibody (Abcam, Cambridge, MA) or anti-CD169 antibody (Biorbyt, Cambridge, UK). Then, the sections were rinsed with PBST three times, and incubated with biotinylated goat anti-rabbit IgG secondary antibody (Beijing Zhongshan Golden Bridge Biotechnology CO., Ltd., Beijing, China) at room temperature for 30 min. After rinsing three times with PBST, the sections were incubated with avidin-HRP at room temperature for 30 min. The sections were washed 5 times with PBST, and immunodetected by using 3,3’-diaminobenzidine (DAB). Finally, all sections were counterstained with hematoxylin and mounted in neutral resin, and observed at 40× magnification with an Olympus CK 40 microscope. The ratio of positive cells containing CD11c, CD64, and CD169 was analyzed by using Image-Pro® Plus (v 6.0, Media Cybemetics Inc., MD).

### Analysis of Cytokine Concentrations in Colon

The colons (about 100 mg) were added into ice-cold PBS, shattered at 4°C, and centrifuged at 5,000×*g* for 15 min at 4°C. Then, the supernatants were collected. The levels of interleukin-1β (IL-1β), IL-4, IL-10, IL-17, and IL-35 in colon were measured with ELISA kits from Shanghai Xinle Co., Ltd. (Shanghai, China).

### The mRNA Expression of Some Genes in Colon

Total RNA in colon was extracted with TRIZOL reagent [TaKaRa Biotechnology (Dalian) Co., Ltd, Dalian, China] according to the manufacturer’s instructions. The RNA level was determined with DU 640 UV spectrophotometer detection (Beckman Coulter Inc., Fullerton, CA), and OD_260_:OD_280_ ratio was 1.8–2.0. The RNA quality in colonic samples was evaluated with 1% agarose gel electrophoresis. Then, in all samples, the cDNA was synthesized by using RT Reagents [TaKaRa Biotechnology (Dalian) Co., Ltd, Dalian, China] according to the manufacturer’s instructions. The gene primers of this study, listed in **Supplemental Table 2**, were purchased from TaKaRa Biotechnology (Dalian) Co., Ltd (Dalian, China). The mRNA expression of zonula occluden 1 (*ZO-1*), mucin 2 (*MUC2*), *Claudin-1*, *TLR4*, *MyD88*, and *NF-κB* in colon was determined by real-time quantitative PCR with SYBR Premix Ex Taq reagents (TaKaRa Biotechnology (Dalian) Co., Ltd, Dalian, China) and CFX-96 Real-Time PCR Detection System (Bio-Rad Laboratories, Richmond, CA) as described previously (17). To correct the variance in the amount of RNA input of reaction, β-actin was used as the reference gene. Then, the relative mRNA expression of target genes was acquired with the previous method (18).

### Statistical Analysis

Statistical analyses were performed with SPSS 20.0 (Statistical Product and Service Solutions, Inc., USA). (i) Cell-culture trial. Data were analyzed by using one-way ANOVA, followed by Duncan’s Multiple Range test. (ii) Animal trial. The growth performance data of rats before DSS challenge, and the DAI data were analyzed with the unpaired *t*-test. The other data were analyzed as a 2 × 2 factorial with the general linear model procedures. The model factors included the effect of l-isoleucine administration, DSS challenge and their interaction. All data were indicated as means with their SEs. The *P*-value less than 0.05 was considered statistical significance while the *P*-value less than 0.10 was deemed a statistical tendency.

## Results

### The Effect of l-Isoleucine and/or TNF-α on Cell Viability in IEC-18 Cells

**Figure 1:**
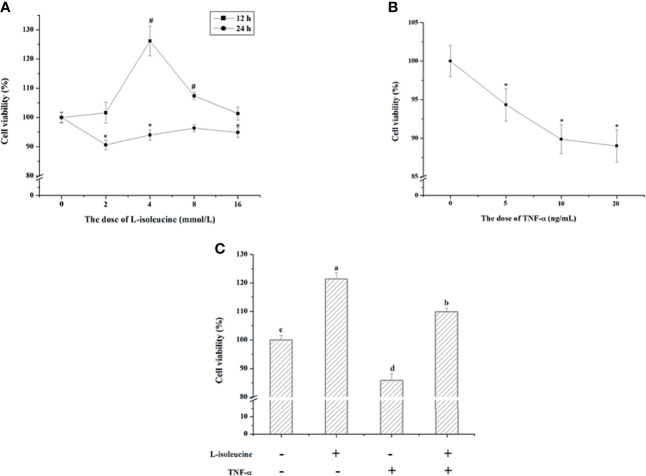
The effect of l-isoleucine and/or TNF-α on cell viability in IEC-18 cells. **(A)** The dosage and duration of l-isoleucine treatment in IEC-18 cells (n = 10 or 12). **(B)** The dosage of TNF-α treatment in IEC-18 cells (n = 12). **(C)** L-isoleucine treatment relieved the negative effect of TNF-α on cell viability in IEC-18 cells (n = 8). Values are means ± SE. Values with different letters are significantly different (*P <*0.05), #, * Compared with 0 mmol/L i isoleucine or 0 ng/mL TNF-α in the same duration, values mean significant difference (*P* < 0.05).

IEC-18 cells were exposed to different levels (0, 2, 4, 8, and 16 mmol/L) of l-isoleucine for 12 and 24 h, and exposed to different concentrations (0, 5, 10, and 20 ng/ml) of TNF-α for 24 h. During 12 h, the IEC-18 cell viability was increased by 4 and 8 mmol/L of l-isoleucine, and 4 mmol/L l-isoleucine treatment improved 26.17% viability (*P <*0.05, **Figure 1A**). After 24 h incubation, 2, 4, and 16 mmol/L l-isoleucine decreased the IEC-18 cell viability (*P <*0.05, **Figure 1A**). The dose and duration of l-isoleucine exposure were 4 mmol/L and 12 h, respectively. Following 24 h exposure, 5, 10, and 20 ng/ml of TNF-α reduced the IEC-18 cell viability (*P <*0.05, **Figure 1B**). The dose of TNF-α exposure was 10 ng/ml. As shown in **Figure 1C**, the viability of IEC-18 cells was also decreased by TNF-α exposure, but after 4 mmol/L l-isoleucine treatment for 12 h, the reduction of IEC-18 cell viability induced by TNF-α challenge was relieved (*P <*0.05).

### Growth Performance and DAI in Rats

**Figure 2:**
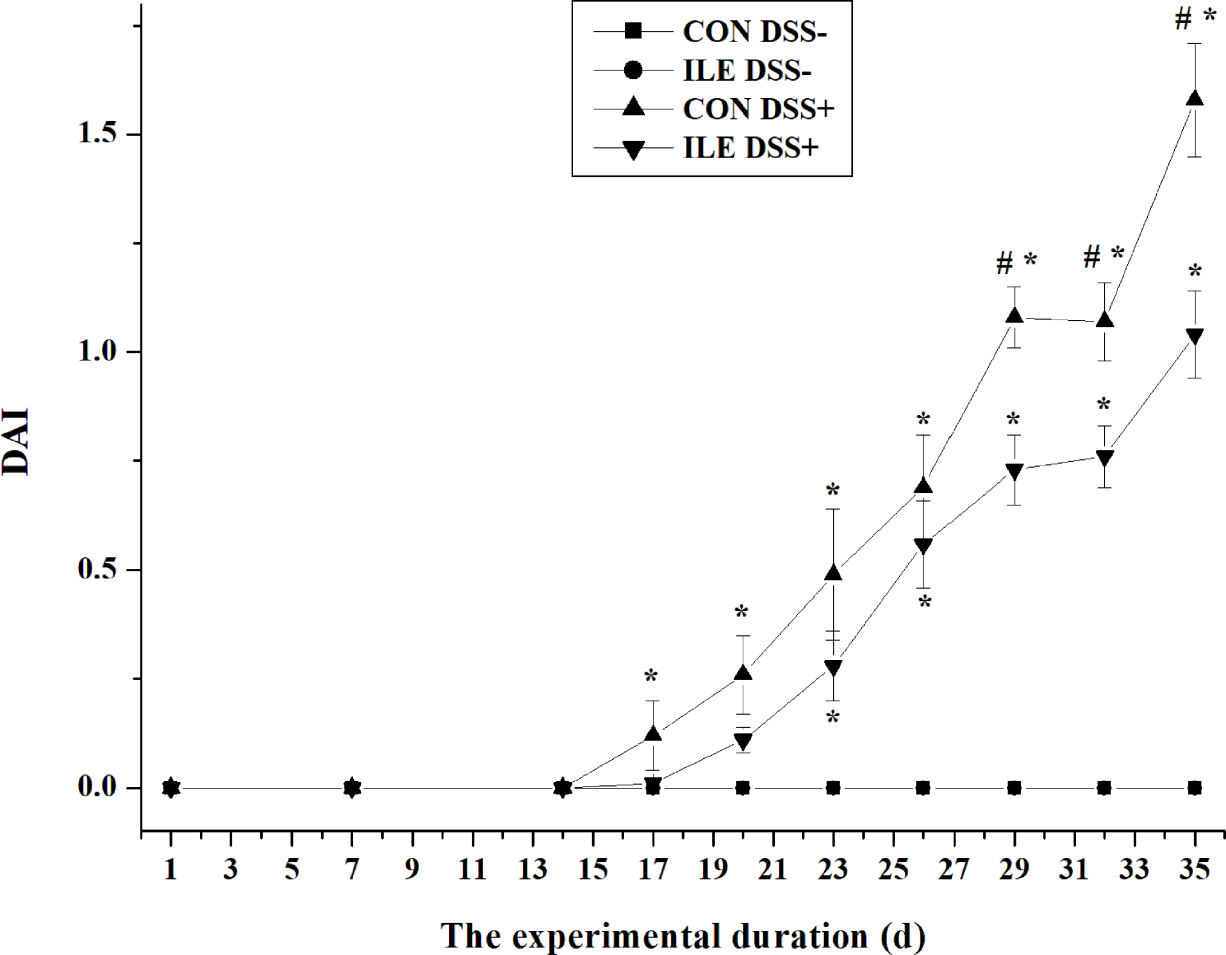
The effect of l-isoleucine administration on disease activity index (DAI) in the normal and DSS-challenge rats. DSS−, drinking the ultrapure water; DSS+, drinking the ultrapure water with DSS; CON, l-alanine-supplemented diet; ILE, l-isoleucine-supplemented diet. Values are means ± SE (n = 11). ^*^Compared with the group of CON DSS−, values mean significant difference (*P <* 0.05). ^#^Compared with the group of ILE DSS+, values mean significant difference (*P <* 0.05).

During the first two weeks, dietary l-isoleucine supplementation increased ADG (*P <*0.05), and decreased F/G (*P <*0.05), but did not affect ADFI and DAI (*P >*0.05) in rats (**Table 1** and **Figure 2**). From 15 to 35 d, DSS challenge impaired growth performance (*P <*0.05) and increased DAI (*P <*0.05), and supplementing l-isoleucine in diet improved ADG (*P <*0.05), ADFI (*P* = 0.07) and F/G (*P <*0.05) in rats (**Table 1** and **Figure 2**). Additionally, in the DSS-challenge rats, l-isoleucine administration alleviated the negative effect of DSS infusion on growth performance (**Table 1**), and delayed the DSS-induced DAI (**Figure 2**).

**Table 1:** Effects of dietary 1.00% l-isoleucine supplementation and/or DSS challenge on growth performance of rats.

	DSS−		DSS+		*P-*value			
	CON	ILE	CON	ILE	ILE	DSS		ILE × DSS
1–14 d								
ADG (g)	6.13 ± 0.09	6.50 ± 0.12			<0.05			
ADFI (g)	15.44 ± 0.07	15.53 ± 0.13			0.55			
F/G	2.53 ± 0.03	2.40 ± 0.05			<0.05			
15–35 d								
ADG (g)	6.40 ± 0.14^b^	7.36 ± 0.16^a^	5.28 ± 0.18^d^	5.93 ± 0.24^c^	<0.05		<0.05	0.40
ADFI (g)	24.59 ± 0.07^a^	24.58 ± 0.10^a^	23.26 ± 0.15^c^	23.66 ± 0.08^b^	0.07		<0.05	0.05
F/G	3.86 ± 0.08^b^	3.36 ± 0.08^c^	4.44 ± 0.14^a^	4.04 ± 0.16^b^	<0.05		<0.05	0.68

DSS−, drinking the ultrapure water; DSS+, drinking the ultrapure water with DSS; CON, l-alanine-supplemented diet; ILE, l-isoleucine-supplemented diet; ADG, average daily gain; ADFI, average daily feed intake; F/G, feed conversion.

^a,b,c,d^In the same row, values with different letter superscripts mean significant difference (P <0.05).

### The Serum Levels of Free Amino Acids and Urea Nitrogen in Rats

DSS challenge increased serum levels of serine (*P <*0.05), glutamate (*P <*0.05), glycine (*P <*0.05), alanine (*P <*0.05), arginine (*P <*0.05), and urea nitrogen (*P* = 0.08), and decreased serum valine level (*P <*0.05) in rats (**Table 2**). Dietary l-isoleucine supplementation enhanced serum concentrations of threonine (*P <*0.05), isoleucine (*P <*0.05), lysine (*P* = 0.06) and arginine (*P <*0.05), and reduced serum glutamate (*P <*0.05), valine (*P <*0.05), and urea nitrogen (*P <*0.05) levels in rats (**Table 2**). Moreover, in the DSS-challenge rats, l-isoleucine administration attenuated the effect of DSS challenge on serum glutamate and urea nitrogen levels, and further enhanced serum arginine concentration (*P <*0.05, **Table 2**).

**Table 2:** Effects of dietary 1.00% l-isoleucine supplementation and/or DSS Challenge on serum levels of free amino acids and urea nitrogen in rats.

	DSS−		DSS+		*P-*value		
	CON	ILE	CON	ILE	ILE	DSS	ILE × DSS
Free amino acids (nmol/L)							
Aspartate	8.12 ± 0.30	6.77 ± 0.67	7.85 ± 0.50	7.80 ± 0.55	0.19	0.48	0.22
Threonine	52.81 ± 1.55^b^	61.97 ± 1.94^a^	55.74 ± 3.35^ab^	56.37 ± 1.18^ab^	<0.05	0.54	0.06
Serine	32.66 ± 1.03^b^	33.51 ± 1.63^ab^	37.40 ± 1.41^a^	35.95 ± 1.61^ab^	0.83	<0.05	0.43
Glutamate	22.86 ± 1.76^b^	20.47 ± 1.69^b^	30.90 ± 2.62^a^	22.49 ± 2.02^b^	<0.05	<0.05	0.15
Glycine	29.29 ± 1.38	30.74 ± 1.90	33.77 ± 1.80	33.76 ± 1.55	0.67	<0.05	0.67
Alanine	70.58 ± 0.97^b^	65.21 ± 1.88^b^	78.49 ± 3.79^a^	71.30 ± 2.76^ab^	0.73	<0.05	<0.05
Valine	23.50 ± 0.64^a^	20.61 ± 0.53^b^	19.49 ± 1.10^b^	18.57 ± 0.80^b^	<0.05	<0.05	0.23
Cysteine	0.47 ± 0.13	0.30 ± 0.08	0.35 ± 0.09	0.39 ± 0.14	0.57	0.91	0.37
Methionine	9.27 ± 0.22	8.31 ± 0.35	8.27 ± 0.47	8.44 ± 0.40	0.30	0.25	0.14
Isoleucine	13.43 ± 0.58^b^	31.28 ± 1.21^a^	12.49 ± 0.40^b^	33.29 ± 1.99^a^	<0.05	0.66	0.24
Leucine	28.35 ± 1.08	27.05 ± 0.99	27.27 ± 1.09	25.82 ± 1.41	0.24	0.32	0.95
Tyrosine	13.22 ± 0.74	12.86 ± 0.43	12.73 ± 0.74	12.34 ± 1.69	0.71	0.63	0.99
Phenylalanine	15.98 ± 0.73	17.42 ± 0.57	16.13 ± 0.43	16.71 ± 0.66	0.11	0.65	0.49
Tryptophan	4.19 ± 1.04	5.28 ± 1.31	4.78 ± 0.62	4.88 ± 0.99	0.56	0.93	0.63
Lysine	48.07 ± 1.20	54.86 ± 3.18	51.48 ± 1.43	54.80 ± 3.64	0.06	0.52	0.51
Histidine	12.60 ± 0.48	12.56 ± 0.50	11.74 ± 1.14	12.68 ± 0.67	0.55	0.62	0.51
Arginine	10.97 ± 4.83^c^	23.73 ± 6.02^bc^	35.94 ± 5.16^b^	54.39 ± 7.09^a^	<0.05	<0.05	0.63
Proline	60.93 ± 2.74	68.31 ± 1.31	67.27 ± 2.77	60.67 ± 3.59	0.89	0.81	<0.05
Urea nitrogen (mmol/L)	9.86 ± 0.38^ab^	8.80 ± 0.30^c^	10.58 ± 0.34^a^	9.23 ± 0.19^bc^	<0.05	0.08	0.63

DSS−, drinking the ultrapure water; DSS+, drinking the ultrapure water with DSS; CON, l-alanine-supplemented diet; ILE, l-isoleucine-supplemented diet.

^a,b,c^ In the same row, values with different letter superscripts mean significant difference (P < 0.05).

### Colonic Length and Histopathology

**Figure 3:**
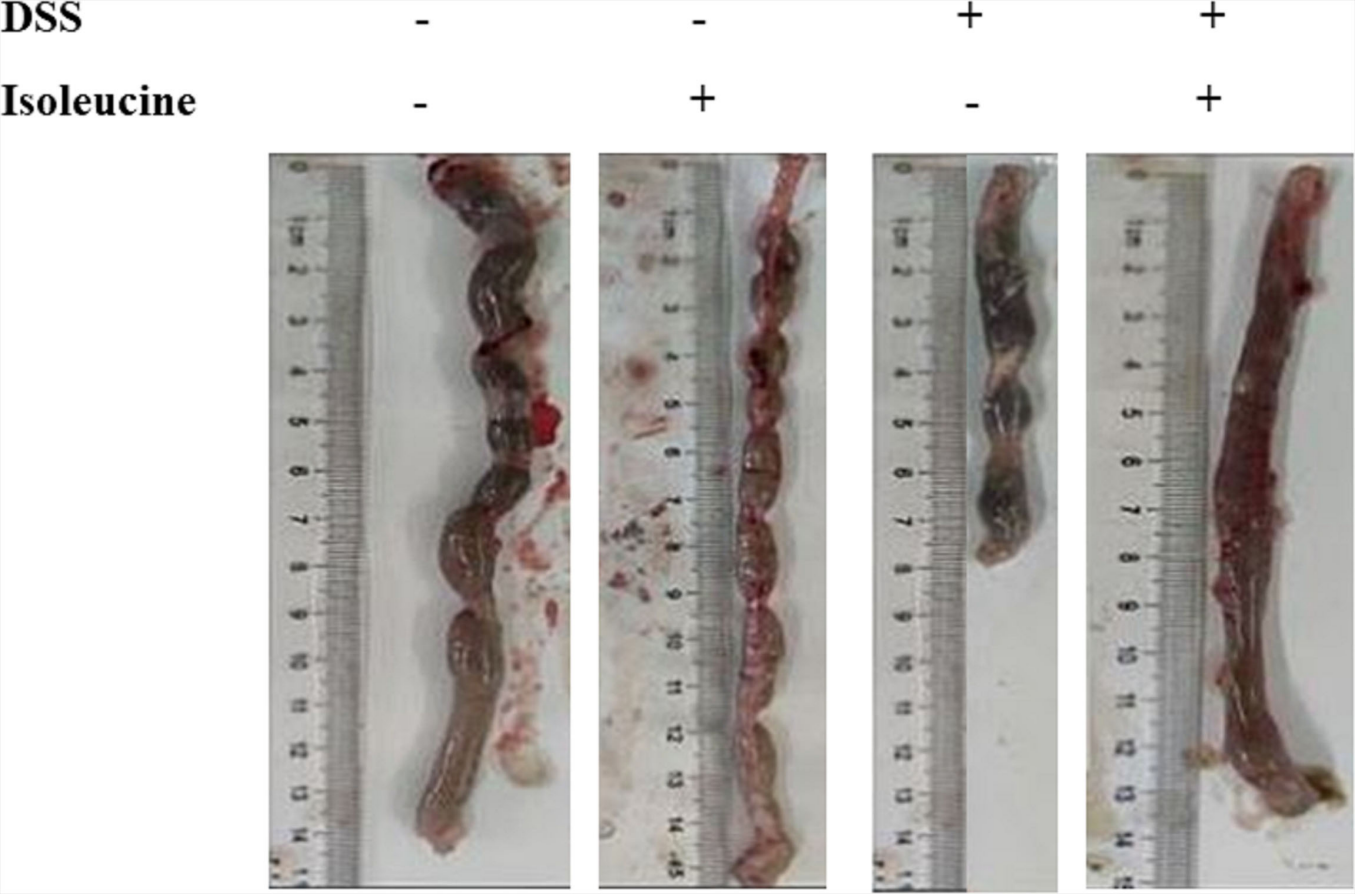
The effect of l-isoleucine administration on colon length in the normal and DSS-challenge rats. DSS, drinking the ultrapure water with DSS; isoleucine, l-isoleucine-supplemented diet.

**Figure 4:**
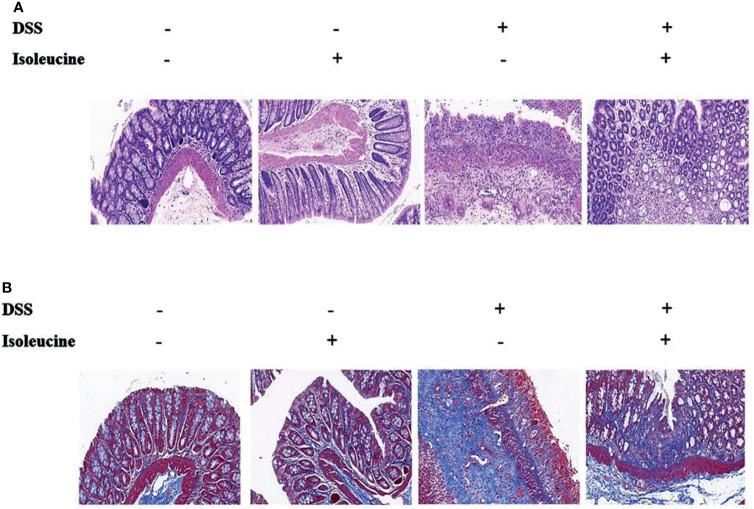
The colonic histopathology in the normal and DSS-challenge rats. **(A)** Colon stained with hematoxylin–eosin (H&E); **(B)** Colon stained with Masson’s trichrome (MT). DSS, drinking the ultrapure water with DSS; isoleucine, l-isoleucine-supplemented diet.

Colonic length in rats was shortened by DSS challenge (*P <*0.05, **Table 3**, **Figure 3**). Dietary l-isoleucine supplementation relieved the negative effect of DSS challenge on colonic length in the DSS-challenge rats (*P <*0.05, **Table 3** and **Figure 3**). There were no pathological changes in the non-challenge rats (**Figure 4**). In the colon of DSS-challenge rats fed l-alanine-supplemented diet, epithelial structure was damaged, mucous layer had the extensive ulcer and sizable infiltration of lymphocytes and neutrophilic granulocytes, muscular layer also had infiltration of lymphocytes and neutrophilic granulocytes, and there were the extensive hyperplasia of collagenous fiber (**Figure 4**). In the colon of DSS-challenge rats fed with l-isoleucine-supplemented diet, epithelial structure was normal, mucous layer was basically integral, but mucous layer had infiltration of some lymphocytes and neutrophilic granulocytes, and there was the weak hyperplasia of collagenous fiber (**Figure 4**).

**Table 3:** Effects of dietary 1.00% l-isoleucine supplementation and/or DSS challenge on colon length and the mRNA expression of barrier-related genes of colon in rats.

	DSS−		DSS+		*P-*value		
	CON	ILE	CON	ILE	ILE	DSS	ILE × DSS
Colon length (cm)	14.13 ± 0.43^a^	15.02 ± 0.41^a^	10.89 ± 0.55^c^	12.61 ± 0.32^b^	<0.05	<0.05	0.35
The mRNA expression of barrier-related genes in colon							
*ZO-1*	1.00 ± 0.14^a^	1.03 ± 0.33^a^	0.15 ± 0.01^b^	0.88 ± 0.17^a^	<0.05	<0.05	0.68
*MUC2*	1.00 ± 0.22	1.05 ± 0.33	1.01 ± 0.06	0.92 ± 0.27	0.93	0.81	0.77
*Claudin-1*	1.00 ± 0.20^ab^	1.38 ± 0.17^a^	0.61 ± 0.11^b^	1.24 ± 0.36^a^	<0.05	0.18	0.52

DSS−, drinking the ultrapure water; DSS+, drinking the ultrapure water with DSS; CON, l-alanine-supplemented diet; ILE, l-isoleucine-supplemented diet; ZO-1, zonula occluden 1; MUC2, mucin 2.

^a,b,c^ In the same row, values with different letter superscripts mean significant difference (P <0.05).

### The mRNA Expression of Barrier-Related Genes in Colon

As shown in **Table 3**, the mRNA expression of *ZO-1* was decreased by DSS challenge (*P <*0.05), but dietary l-isoleucine supplementation increased the *ZO-1* mRNA expression in the colon of rats (*P <*0.05). In the DSS-challenge rats, l-isoleucine administration could improve the *ZO-1* and *Claudin-1* mRNA expression of colon (*P <*0.05, **Table 3**).

### Enterocyte Apoptosis and Cell Cycle in Colon

DSS challenge increased the early-stage, late-stage and total apoptotic cell percentages, enhanced the ratio of G_0_/G_1_ phase cells, and decreased the proportion of S phase cells in the colonic epithelium of rats (*P <*0.05, **Tables 4**, **5**, and **Supplementary Figures 1**, **2**). Dietary l-isoleucine supplementation reduced late-stage and total apoptotic cell percentages, and the proportion of G_0_/G_1_ phase cells, but elevated the ratio of S phase cells in the colonic epithelium of rats (*P <*0.05, **Tables 4**, **5**, and **Supplementary Figures 1** and **2**). Furthermore, in the DSS-challenge rats, supplementing l-isoleucine in diet relieved the effect of DSS challenge on the late-stage and total apoptotic cell percentages in the colonic epithelium of rats (*P <*0.05, **Table 4** and **Supplementary Figure 1**).

**Table 4:** Effects of dietary 1.00% l-isoleucine supplementation and/or DSS challenge on enterocyte apoptosis in colon of rats (%).

	DSS−		DSS+		*P-*value		
	CON	ILE	CON	ILE	ILE	DSS	ILE × DSS
Early-stage apoptotic cells	1.44 ± 0.35	1.59 ± 0.15	2.72 ± 0.78	2.94 ± 0.46	0.71	<0.05	0.94
Late-stage apoptotic cells	1.11 ± 0.22^b^	0.25 ± 0.06^b^	4.69 ± 1.30^a^	1.93 ± 0.78^b^	<0.05	<0.05	0.25
Total apoptotic cells	2.54 ± 0.18^c^	1.84 ± 0.12^c^	7.41 ± 0.64^a^	4.87 ± 0.59^b^	<0.05	<0.05	0.08

DSS−, drinking the ultrapure water; DSS+, drinking the ultrapure water with DSS; CON, l-alanine-supplemented diet; ILE, l-isoleucine-supplemented diet.

^a,b,c^In the same row, values with different letter superscripts mean significant difference (P <0.05).

**Table 5:** Effects of dietary 1.00% l-isoleucine supplementation and/or DSS challenge on cell cycle in colon of rats (%).

	DSS−		DSS+		*P-*value		
	CON	ILE	CON	ILE	ILE	DSS	ILE × DSS
G_0_/G_1_ phase cells	86.54 ± 2.28^a^	71.80 ± 4.34^b^	90.37 ± 0.55^a^	88.50 ± 0.16^a^	<0.05	<0.05	<0.05
S phase cells	7.66 ± 1.85^b^	20.48 ± 3.65^a^	4.17 ± 0.51^b^	5.73 ± 0.10^b^	<0.05	<0.05	<0.05
G_2_ + M phase cells	5.81 ± 1.14	7.72 ± 0.80	5.46 ± 0.33	5.77 ± 0.24	0.17	0.15	0.30

DSS−, drinking the ultrapure water; DSS+, drinking the ultrapure water with DSS; CON, l-alanine-supplemented diet; ILE, l-isoleucine-supplemented diet.

^a,b^In the same row, values with different letter superscripts mean significant difference (P <0.05).

### Inflammation-Related Markers in Colon

**Figure 5:**
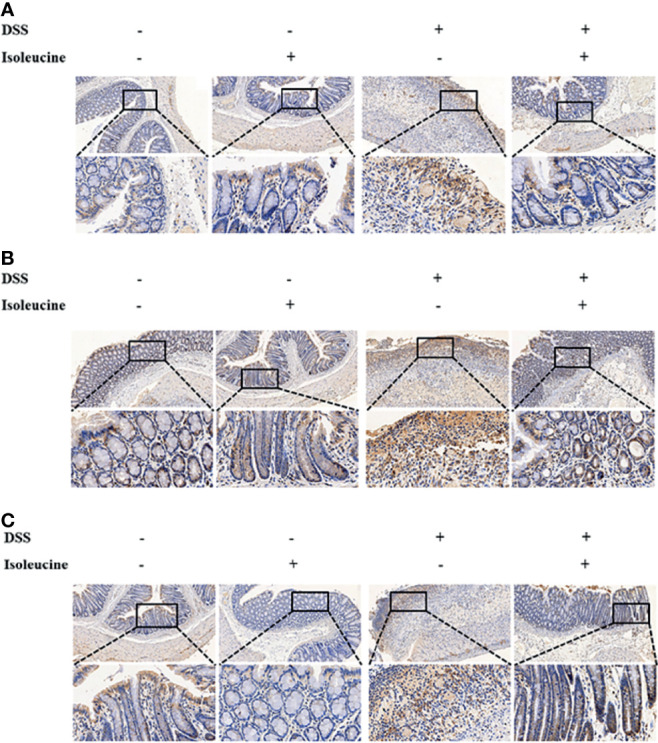
The effect of l-isoleucine administration on the positive cells containing CD11c **(A)**, CD64 **(B)**, and CD169 **(C)** in the colonic mucosa of normal and DSS-challenge rats. DSS, drinking the ultrapure water with DSS; isoleucine, l-isoleucine-supplemented diet.

DSS challenge elevated the ratio of positive cells containing CD11c, CD64, and CD169 (*P <*0.05), but l-isoleucine administration reduced the ratio of positive cells containing CD11c (*P <*0.05), CD64 (*P <*0.05), and CD169 (*P* = 0.08) in the colonic mucosa of rats (**Table 6** and **Figure 5**). Moreover, in the DSS-challenge rats, supplementing l-isoleucine in diet relieved the DSS-induced enhancement of the ratio of CD11c-, CD64-, and CD169-positive cells in the colonic mucosa of rats (*P <*0.05, **Table 6** and **Figure 5**).

**Table 6:** Effects of dietary 1.00% l-isoleucine supplementation and/or DSS challenge on the ratio of positive cells containing CD11c, CD64 and CD169 in the colon of rats.

	DSS−		DSS+		*P-*value		
	CON	ILE	CON	ILE	ILE	DSS	ILE × DSS
CD11c	6.67 ± 0.33^c^	4.33 ± 0.33^d^	21.33 ± 0.88^a^	11.67 ± 0.33^b^	<0.05	<0.05	<0.05
CD64	46.67 ± 0.33^c^	34.33 ± 0.33^d^	81.33 ± 0.88^a^	62.33 ± 0.67^b^	<0.05	<0.05	<0.05
CD169	9.33 ± 2.85^c^	11.67 ± 2.33^c^	42.67 ± 1.20^a^	32.33 ± 0.67^b^	0.08	<0.05	<0.05

DSS−, drinking the ultrapure water; DSS+, drinking the ultrapure water with DSS; CON, l-alanine-supplemented diet; ILE, l-isoleucine-supplemented diet; MPO, myeloperoxidase.

^a,b,c,d^In the same row, values with different letter superscripts mean significant difference (P <0.05).

### The Level of Cytokines in Colon

The effect of DSS challenge and/or l-isoleucine administration on the level of cytokines in the colon of rats is shown in **Table 7**. DSS challenge increased the levels of IL-1β and IL-17, and decreased the IL-35 concentration in the colon of rats (*P <*0.05). L-isoleucine administration reduced the IL-1β and IL-17 concentrations, and enhanced the level of IL-4 in the colon of rats (*P <*0.05). In addition, the effect of DSS challenge on the levels of IL-1β, IL-4, and IL-17 in the colon of rats could be alleviated by dietary l-isoleucine supplementation (*P <*0.05).

**Table 7:** Effects of dietary 1.00% l-isoleucine supplementation and/or DSS challenge on cytokine levels in colon of rats (ng/mg protein).

	DSS−		DSS+		*P-*value		
	CON	ILE	CON	ILE	ILE	DSS	ILE × DSS
IL-1β	2.43 ± 0.29^b^	1.99 ± 0.14^b^	4.00 ± 0.39^a^	2.82 ± 0.31^b^	<0.05	<0.05	0.24
IL-4	20.56 ± 1.39^b^	26.96 ± 1.39^a^	15.09 ± 0.66^c^	27.34 ± 1.14^a^	<0.05	0.15	0.10
IL-10	67.66 ± 3.04	60.23 ± 3.07	56.21 ± 2.26	73.11 ± 2.78	0.10	0.80	<0.05
IL-17	14.16 ± 0.87^bc^	11.53 ± 0.75^c^	26.05 ± 1.59^a^	16.60 ± 1.26^b^	<0.05	<0.05	<0.05
IL-35	99.38 ± 6.78^a^	82.13 ± 6.39^b^	77.83 ± 2.27^b^	76.64 ± 4.89^b^	0.10	<0.05	0.15

DSS−, drinking the ultrapure water; DSS+, drinking the ultrapure water with DSS; CON, l-alanine-supplemented diet; ILE, l-isoleucine-supplemented diet; IL, interleukin.

^a,b,c^In the same row, values with different letter superscripts mean significant difference (P <0.05).

### The mRNA Expression of *TLR4*, *MyD88*, and *NF-κB* in Colon

DSS challenge stimulated the mRNA expression of *TLR4*, *MyD88*, and *NF-κB* in the colon of rats (*P <*0.05, **Table 8**). Dietary l-isoleucine supplementation inhibited the mRNA expression of *TLR4*, *MyD88*, and *NF-κB* in the colon of rats (*P <*0.05, **Table 8**). In the colon of DSS-challenge rats, the upregulation of *TLR4*, *MyD88*, and *NF-κB* mRNA expression was also relieved by l-isoleucine administration (*P <*0.05, **Table 8**).

**Table 8:** Effects of dietary 1.00% l-isoleucine supplementation and/or DSS challenge on the relative expression of inflammation-related genes in colon of rats.

	DSS−		DSS+		*P-*value		
	CON	ILE	CON	ILE	ILE	DSS	ILE × DSS
TLR-4	1.00 ± 0.34^b^	0.91 ± 0.29^b^	3.17 ± 0.62^a^	0.84 ± 0.43^b^	<0.05	<0.05	<0.05
MyD88	1.00 ± 0.28^b^	1.94 ± 0.27^b^	50.59 ± 7.70^a^	4.16 ± 0.63^b^	<0.05	<0.05	<0.05
NF-κB	1.00 ± 0.31^b^	0.87 ± 0.19^b^	78.13 ± 14.13^a^	1.17 ± 0.38^b^	<0.05	<0.05	<0.05

DSS−, drinking the ultrapure water; DSS+, drinking the ultrapure water with DSS; CON, l-alanine-supplemented diet; ILE, l-isoleucine-supplemented diet; TLR4, toll-like receptor 4; MyD88, myeloid differentiation primary response gene 88; NF-κB, nuclear factor-kappa B.

^a,b^In the same row, values with different letter superscripts mean significant difference (P <0.05).

## Discussion

Isoleucine, known as one of branched chain amino acids, has many physiological functions, especially immunity (8, 9, 12). Many previous studies have also shown that isoleucine administration may regulate the generation of immunoreactive substances (such as cytokines) (12). We also found that l-isoleucine administration could influence the mRNA expression and concentration of inflammation-related cytokines of ileal mucosa in weaned piglets (13). In this study, l-isoleucine treatment alleviated the negative effect of TNF-α on cell viability in IEC-18 cells, and then dietary l-isoleucine supplementation also improved growth performance, and decreased DAI, colon damage and inflammation in the DSS-challenge rats. This was the main finding of our study.

IBD, especially colitis, is one of typical digestive-tract diseases that influence human health (2). This makes IBD be a focus of clinical researches. Oral infusion of DSS in rodent models may establish colitis, which leads to the decrease of growth, the increase of DAI (namely, diarrhea and stool containing blood), the damage of colon, the dysfunction of colonic morphology, and inflammation in colon (3, 4, 16). In the present study, drinking the DSS-supplemented (4%) water also induced the similar symptom, and upregulated some inflammatory markers in rats. Therefore, it is proposed that DSS-induced colitis model was successfully set up.

Supplementing l-isoleucine in diet may increase growth performance of some livestock and fish (such as pigs, laying hens, and juvenile Jian carp) (19–23). Our recent study also showed that l-isoleucine administration improved growth in the normal and RV-infected piglets (13). In the current study, dietary l-isoleucine supplementation ameliorated ADG and F/G ratio of rats before DSS challenge, and also attenuated the negative effect on ADFI, ADG and F/G ratio under the condition of DSS challenge. Besides, l-isoleucine administration assuaged the increasing serum urea nitrogen level that was induced by drinking the DSS-supplemented water. Urea nitrogen in blood is usually considered as an indirect marker for protein metabolism in the whole body (24, 25). These illustrated that DSS challenge impaired protein metabolism in rats, which could be improved by dietary l-isoleucine supplementation. Thus, supplementing l-isoleucine in diet increasing growth performance of rats was potentially associated with the enhancement of protein metabolism in whole body.

Amino acids, especially some functional amino acids, play an important role in maintaining physiological functions. Glutamate may be the energy source of gut epithelial cells, which maintains the gut health (26). Arginine can relieve the inflammation in the whole body (27). In our study, l-isoleucine administration decreased serum glutamate level, and increased serum arginine level in the DSS-challenge rats. These illustrated that dietary l-isoleucine supplementation could promote the energy consumption of gut and the generation of anti-inflammatory substance, and then improve gut health and inflammatory reaction.

The colitis embodies colonic damage, which is the main reason that DSS challenge induces the decrease of growth and health (2). In this study, l-isoleucine administration, to some extent, alleviated the DSS-induced dysfunction in colon, namely, the increase of colon length, the increasing integration of epithelial structure and mucous layer, the reducing infiltration of some lymphocytes and neutrophilic granulocytes in mucous layer, and the weak hyperplasia of collagenous fiber. Moreover, we also found that supplementing l-isoleucine in diet could improve the *ZO-1* and *Claudin-1* mRNA expression in the colon of DSS-challenge rats, which is similar with the results of piglets in our previous study (28). Therefore, dietary l-isoleucine supplementation improving growth of DSS-challenge rats could be also derived from the decrease of colonic damage.

The cell death from apoptosis and cell proliferation play an important role for the integration of intestinal mucosa structure (29, 30). To further analyze the possible mechanism of l-isoleucine regulating colonic mucosa structure, we measured cell apoptosis and cell cycle in the colonic mucosa of rats. In this study, drinking the DSS-supplemented water aggravated the percentage of apoptotic cells (namely, early-stage, late-stage and total apoptotic cells) and impaired the ratio of G_0_/G_1_ and S phase cells in the colonic mucosa of rats, while l-isoleucine administration only assuaged the increasing percentage of late-stage and total apoptotic cells, and did not affect the percentage of early-stage apoptotic cells and cell cycle in the colonic mucosa of DSS-challenge rats. These illustrated that dietary l-isoleucine supplementation improving DSS-induced colonic damage could mainly be related to the relief of upregulating late-stage apoptosis in the colonic mucosa of rats.

Inflammation is one of the reasons that affect cell death (such as apoptosis) (31, 32). Thus, dietary l-isoleucine supplementation relieving cell apoptosis could be due to the decrease of DSS-induced inflammation.

CD11c, known as the specific marker in dendritic cells, is a transmembrane glycoprotein, and is relative with some immune reaction (namely, inflammation) (33). CD64 is the receptor of immunoglobulin G in dendritic cells, monocytes and macrophages, and plays an important role for the production of inflammatory cytokines (34). CD169 exists in the surface of some specific macrophage subgroups, and is beneficial for macrophage recognition, and then regulates inflammatory reaction (35). In this study, l-isoleucine administration downregulated the increasing ratio of positive cells containing CD11c, CD64, and CD169 in the colon of DSS-challenge rats, which was consistent with the results of histopathology in colon.

T helper cells (Th cells, namely, Th1, Th2, and Th17 cells) and regulatory T cells (Treg cells) are the special subset of T cells, and the balance of Th1/Th2 and Th17/Treg plays a critical role for regulating inflammation (36). Their imbalance will be induced in colitis. The subset of T cells can generate pro-inflammatory cytokines (i.e., IL-1β, and IL-17) and anti-inflammatory cytokines (i.e., IL-4, IL-10, and IL-35) (36). In this study, l-isoleucine administration alleviated the effect of DSS challenge on IL-1β, IL-4, and IL-17 levels in the colon of rats. Our recent study has also shown that supplementing l-isoleucine in diet could regulate the production of some inflammation-related cytokines (such as IL-1β, IL-10, and TNF-α) in the ileum of RV-infected piglets (13). There was the different efficiency of l-isoleucine regulating cytokines between the results of two experiments, which could be due to the difference of trial models. Based on the above analysis of inflammatory markers and cytokines, it was proposed that dietary l-isoleucine supplementation might inhibit the DSS-induced inflammation, and then improve cell apoptosis in the colon of rats.

There is the close relationship between the DSS-induced IBD models and the stimulation of TLR4/MyD88/NF-κB pathway that increases the generation of pro-inflammatory cytokines, and the downregulation of this pathway through the different methods which can effectively be used to cure or attenuate the IBD (14, 15). The treatment of l-isoleucine could regulate the *NF-κB* mRNA expression of tissues and cells (13, 37). Our present study also reported that DSS challenge upregulated the mRNA expression of *TLR4*, *MyD88*, and *NF-κB* in the colon of rats while dietary l-isoleucine supplementation effectively inhibited the increasing related-gene mRNA expression that was induced by drinking the DSS-supplementing water. Thus, it was possible that l-isoleucine administration relieving inflammation should be associated with the downregulation of TLR4/MyD88/NF-κB pathway.

In summary, on the basis of l-isoleucine treatment effectively ameliorating the TNF-α-induced inhibition of cell viability in IEC-18 cells, l-isoleucine administration assuaged the negative effect of drinking the DSS-supplementing water on growth performance and colonic health, and reduced, to some extent, the DSS-induced colitis in rats, which could be on account of l-isoleucine treatment relieving the inflammation *via* regulating the TLR4/MyD88/NF-κB pathway. However, the further mechanisms of isoleucine regulating inflammation also needed to be researched in the future. This current study suggests that l-isoleucine might be utilized as the prevention and/or adjuvant therapy of IBD (especially colitis).

## Data Availability Statement

The original contributions presented in the study are included in the article/**Supplementary Material**. Further inquiries can be directed to the corresponding author.

## Ethics Statement

The animal study was reviewed and approved by the Animal Care Advisory Committee of Sichuan Agricultural University.

## Author Contributions

XM, DC, BY, JH, JY, and HY conceived and designed the experiments. XM, RS, QiW, and JY performed the experiments. JL, YL, and JW analyzed the data. HW and QuW contributed reagents/materials/analysis tools. XM, RS, and QiW wrote the paper. All authors contributed to the article and approved the submitted version.

## Funding

This work was supported by the grant from the China Agriculture Research System of MOF and MARA, and the grant from Science and Technology Support Project of Sichuan Province (2021YFYZ0008).

## Conflict of Interest

The authors declare that the research was conducted in the absence of any commercial or financial relationships that could be construed as a potential conflict of interest.

## Publisher’s Note

All claims expressed in this article are solely those of the authors and do not necessarily represent those of their affiliated organizations, or those of the publisher, the editors and the reviewers. Any product that may be evaluated in this article, or claim that may be made by its manufacturer, is not guaranteed or endorsed by the publisher.

## Supplementary Material

Supplementary Figure 1 Scatter diagram of the apoptotic epithelial cells in the colon of normal and DSS-challenge rats. Frames were divided into four quadrants: A-+ represents necrotic cells; A++ represents late-stage apoptotic cells; A– represents normal cells; A+- represents early-stage apoptotic cells. DSS -, drinking the ultrapure water; DSS +, drinking the ultrapure water with DSS; CON, l-alanine-supplemented diet; ILE, l-isoleucine-supplemented diet

Supplementary Figure 2 DNA histogram of the cell cycle in the colonic epithelium cells of normal and DSS-challenge rats. The DNA histogram shows the colonic epithelium cell cycle in the G0/G1 phase (first peak), the G2 + M phase (second peak), and the S phase (region between the first and second peak). DSS -, drinking the ultrapure water; DSS +, drinking the ultrapure water with DSS; CON, l-alanine-supplemented diet; ILE, l-isoleucine-supplemented diet.

Supplementary Figure 3 Graphic abstract.

## References

[1] Mao X Zeng X Qiao S Wu G Li D. Specific Roles of Threonine in Intestinal Mucosal Integrity and Barrier Function. Front Biosci (Elite Ed) (2011) 3:1192–200. doi: 10.2741/e322 21622125

[2] Wang H Chao K Ng SC Bai AH Yu Q Yu J. Pro-Inflammatory miR-223 Mediates the Cross-Talk Between the IL23 Pathway and the Intestinal Barrier in Inflammatory Bowel Disease. Genome Biol (2016) 17(1):58. doi: 10.1186/s13059-016-0901-8 27029486 PMC4815271

[3] Pathak S Grillo AR Scarpa M Brun P D’Incà R Nai L. MiR-155 Modulates the Inflammatory Phenotype of Intestinal Myofibroblasts by Targeting SOCS1 in Ulcerative Colitis. Exp Mol Med (2015) 47(5):e164. doi: 10.1038/emm.2015.21 25998827 PMC4454995

[4] Shi C Liang Y Yang J Xia Y Chen H Han H. MicroRNA-21 Knockout Improve the Survival Rate in DSS Induced Fatal Colitis Through Protecting Against Inflammation and Tissue Injury. PloS One (2013) 8(6):e66814. doi: 10.1371/journal.pone.0066814 23826144 PMC3691313

[5] Kim H Banerjee N Barnes RC Pfent CM Talcott ST Dashwood RH. Mango Polyphenolics Reduce Inflammation in Intestinal Colitis-Involvement of the miR-126/PI3K/AKT/mTOR Axis *In Vitro* and *In Vivo* . Mol Carcinog (2017) 56(1):197–207. doi: 10.1002/mc.22484 27061150 PMC5053910

[6] Qu S Shen Y Wang M Wang X Yang Y. Suppression of miR-21 and miR-155 of Macrophage by Cinnamaldehyde Ameliorates Ulcerative Colitis. Int Immunopharmacol (2019) 67:22–34. doi: 10.1016/j.intimp.2018.11.045 30530166

[7] Xiao Y Dai X Li K Gui G Liu J Yang H. *Clostridium Butyricum* Partially Regulates the Development of Colitis-Associated Cancer Through miR-200c. Cell Mol Biol (2017) 63(4):59–66. doi: 10.14715/cmb/2017.63.4.10 28478805

[8] Nair KS Short KR. Hormonal and Signaling Role of Branched-Chain Amino Acids. J Nutr (2005) 135(6 Suppl):1547S–52S. doi: 10.1093/jn/135.6.1547S 15930467

[9] Nie C He T Zhang W Zhang G Ma X. Branched Chain Amino Acids: Beyond Nutrition Metabolism. Int J Mol Sci (2018) 19(4):954. doi: 10.3390/ijms19040954 PMC5979320 29570613

[10] Ma N Guo P Zhang J He T Kim SW Zhang G. Nutrients Mediate Intestinal Bacteria-Mucosal Immune Crosstalk. Front Immunol (2018) 9:5. doi: 10.3389/fimmu.2018.00005 29416535 PMC5787545

[11] He L Han M Farrar S Ma X. Impacts and Regulation of Dietary Nutrients on Gut Microbiome and Immunity. Protein Pept Lett (2017) 24(5):380–1. doi: 10.2174/092986652405170510214715 28917076

[12] Gu C Mao X Chen D Yu B Yang Q. Isoleucine Plays an Important Role for Maintaining Immune Function. Curr Protein Pept Sci (2019) 20(7):644–51. doi: 10.2174/1389203720666190305163135 30843485

[13] Mao X Gu C Ren M Chen D Yu B He J. L-Isoleucine Administration Alleviates Rotavirus Infection and Immune Response in the Weaned Piglet Model. Front Immuno (2018) 9:1654. doi: 10.3389/fimmu.2018.01654 PMC6054962 30061901

[14] Lin X Liu J. TLR4/MyD88/NF-κb Signaling Pathway and Ulcerative Colitis. Chin J Gastroenterol (2013) 18(4):244–6. doi: 10.3969/j.issn.1008-7125.2013.04.012

[15] Sun W Zhang Z Piao D. Research Progress of NF-κb Signaling Pathway Inhibition on Colitis and Inflammation-Associated Colon Cancer. Med Recapitulate (2020) 26(8):1521–5. doi: 10.3969/j.issn.1006-2084.2020.08.013

[16] Murthy SN Cooper HS Shim H Shah RS Ibrahim SA Sedergran DJ. Treatment of Dextran Sulfate Sodium-Induced Murine Colitis by Intracolonic Cyclosporine. Dig Dis Sci (1993) 38(9):1722–34. doi: 10.1007/BF01303184 8359087

[17] Fan X Hu H Chen D Yu B He J Yu J. Lentinan Administration Alleviates Diarrhea of Rotavirus-Infected Weaned Pigs *via* Regulating Intestinal Immunity. J Anim Sci Biotechnol (2021) 12(1):43. doi: 10.1186/s40104-021-00562-6 33750472 PMC7945689

[18] Livak KJ Schmittgen TD. Analysis of Relative Gene Expression Data Using Real-Time Quantitative PCR and the 2 -ΔΔ CT Method. Methods (2012) 25(4):402–8. doi: 10.1006/meth.2001.1262 11846609

[19] Lordelo MM Gaspar AM Le BL Freire JP. Isoleucine and Valine Supplementation of a Low-Protein Corn-Wheat-Soybean Meal-Based Diet for Piglets: Growth Performance and Nitrogen Balance. J Anim Sci (2008) 86(11):2936–41. doi: 10.2527/jas.2007-0222 18567735

[20] Zhao J Liu Y Jiang J Wu P Chen G Jiang W. Effects of Dietary Isoleucine on Growth, the Digestion and Absorption Capacity and Gene Expression in Hepatopancreas and Intestine of Juvenile Jian Carp (Cyprinus Carpio Var. Jian). Aquaculture (2012) 368-369(1):117–28. doi: 10.1016/j.aquaculture.2012.09.019

[21] Ren M Zhang SH Zeng XF Liu H Qiao SY. Branched-Chain Amino Acids are Beneficial to Maintain Growth Performance and Intestinal Immune-Related Function in Weaned Piglets Fed Protein Restricted Diet. Asian-australas J Anim Sci (2015) 28(12):1742–50. doi: 10.5713/ajas.14.0131 PMC4647083 26580442

[22] Dong XY Azzam MM Zou XT. Effects of Dietary L-Isoleucine on Laying Performance and Immunomodulation of Laying Hens. Poult Sci (2016) 95(10):2297–305. doi: 10.3382/ps/pew163 27118860

[23] Luo Y Zhang X Qin C Jiao N Yin J. Effects of Dietary Isoleucine Level on Growth Performance, Carcass Traits and Meat Quality of Finishing Pigs. Chin J Anim Nutr (2017) 29(6):1884–94. doi: 10.3969/j.issn.1006-267x.2017.06.009

[24] Eggum BO. Blood Urea Measurement as a Technique for Assessing Protein Quality. Br J Nutr (1970) 24(4):983–8. doi: 10.1079/BJN19700101 5484735

[25] Brown JA Cline TR. Urea Excretion in the Pig: An Indicator of Protein Quality and Amino Acid Requirements. J Nutr (1974) 104(5):542–5. doi: 10.1093/jn/104.5.542 4823939

[26] Tomé D. The Roles of Dietary Glutamate in the Intestine. Ann Nutr Metab (2018) 73(suppl 5):15–20. doi: 10.1159/000494777 30508814

[27] Patel VB Preedy VR Rajendram R. L-Arginine in Clinical Nutrition. Switzerland, Cham: Humana Press (2017). doi: 10.1007/978-3-319-26009-9

[28] Mao X Gu C Chen D Yu B He J Zheng P. Effects of Dietary Isoleucine on Ileal Barrier Function of Weaned Piglets Challenged by Rotavirus. Chin J Anim Nutr (2019) 31(12):5493–9. doi: 10.3969/j.issn.1006-267x.2019.12.014

[29] Mao X Xiao X Chen D Yu B He J Chen H. Dietary Apple Pectic Oligosaccharide Improves Gut Barrier Function of Rotavirus-Challenged Weaned Pigs by Increasing Antioxidant Capacity of Enterocytes. Oncotarget (2017) 8(54):92420–30. doi: 10.18632/oncotarget.21367 PMC5696193 29190927

[30] Norbury C Nurse P. Animal Cell Cycles and Their Control. Annu Rev Biochem (1992) 61:441–70. doi: 10.1146/annurev.bi.61.070192.002301 1497317

[31] Lee J Giordano S Zhang J. Autophagy, Mitochondria and Oxidative Stress: Cross-Talk and Redox Signalling. Biochem J (2012) 441:523–40. doi: 10.1042/BJ20111451 PMC3258656 22187934

[32] Patankar JV Becker C. Cell Death in the Gut Epithelium and Implications for Chronic Inflammation. Nat Rev Gastroenterol Hepatol (2020) 17(9):543–56. doi: 10.1038/s41575-020-0326-4 32651553

[33] Vinay DS Kwon BS. CD11c+CD8+ T Cells: Two-Faced Adaptive Immune Regulators. Cell Immunol (2010) 264(1):18–22. doi: 10.1016/j.cellimm.2010.05.010 20620256

[34] Muthas D Reznichenko A Balendran CA Bottcher G Clausen IG Mardh CK. Neutrophils in Ulcerative Colitis: A Review of Selected Biomarkers and Their Potential Therapeutic Implications. Scand J Gastroenterol (2017) 52(2):125–35. doi: 10.1080/00365521.2016.1235224 27610713

[35] O’Neil ASG van den Berg TK Mullen GED. Sialoadhesin-A Macrophage-Restricted Marker of Immunoregulation and Inflammation. Immunology (2013) 138(3):198–207. doi: 10.1111/imm.12042 23181380 PMC3573273

[36] Cseh A Molnár K Pintér P Szalay B Szebeni B Treszl A. Regulatory T Cells and T Helper Subsets in Breast-Fed Infants With Hematochezia Caused by Allergic Colitis. J Pediatr Bastroenterol Nutr (2010) 51(5):675–7. doi: 10.1097/MPG.0b013e3181e85b22 20818268

[37] Fehlbaum P Rao M Zasloff M Anderson GM. An Essential Amino Acid Induces Epithelial Beta-Defensin Expression. Proc Natl Acad Sci USA (2000) 97(23):12723–8. doi: 10.1073/pnas.220424597 PMC18831 11058160

