# Supplementary figures and images for: l-Isoleucine Administration Alleviates DSS-Induced Colitis by Regulating TLR4/MyD88/NF-κB Pathway in Rats
Source: Front Immunol. 2022 Jan 11;12:817583. doi: 10.3389/fimmu.2021.817583 (PMC8787224; doi:10.3389/fimmu.2021.817583)

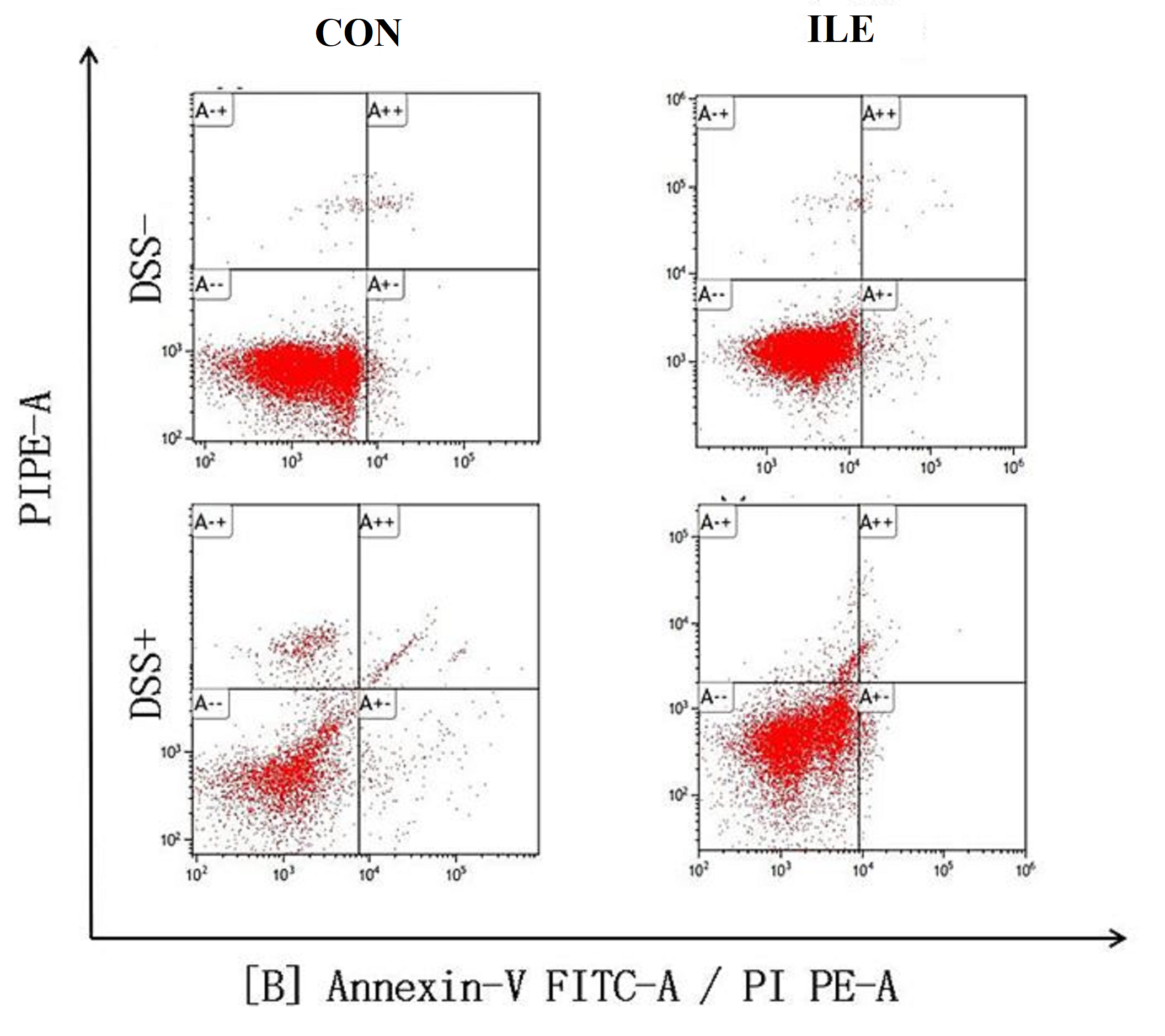

Supplement: Supplementary Figure 1 — Scatter diagram of the apoptotic epithelial cells in the colon of normal and DSS-challenge rats. Frames were divided into four quadrants: A-+ represents necrotic cells; A++ represents late-stage apoptotic cells; A– represents normal cells; A+- represents early-stage apoptotic cells. DSS -, drinking the ultrapure water; DSS +, drinking the ultrapure water with DSS; CON, l-alanine-supplemented diet; ILE, l-isoleucine-supplemented diet [file Image_1.tif]

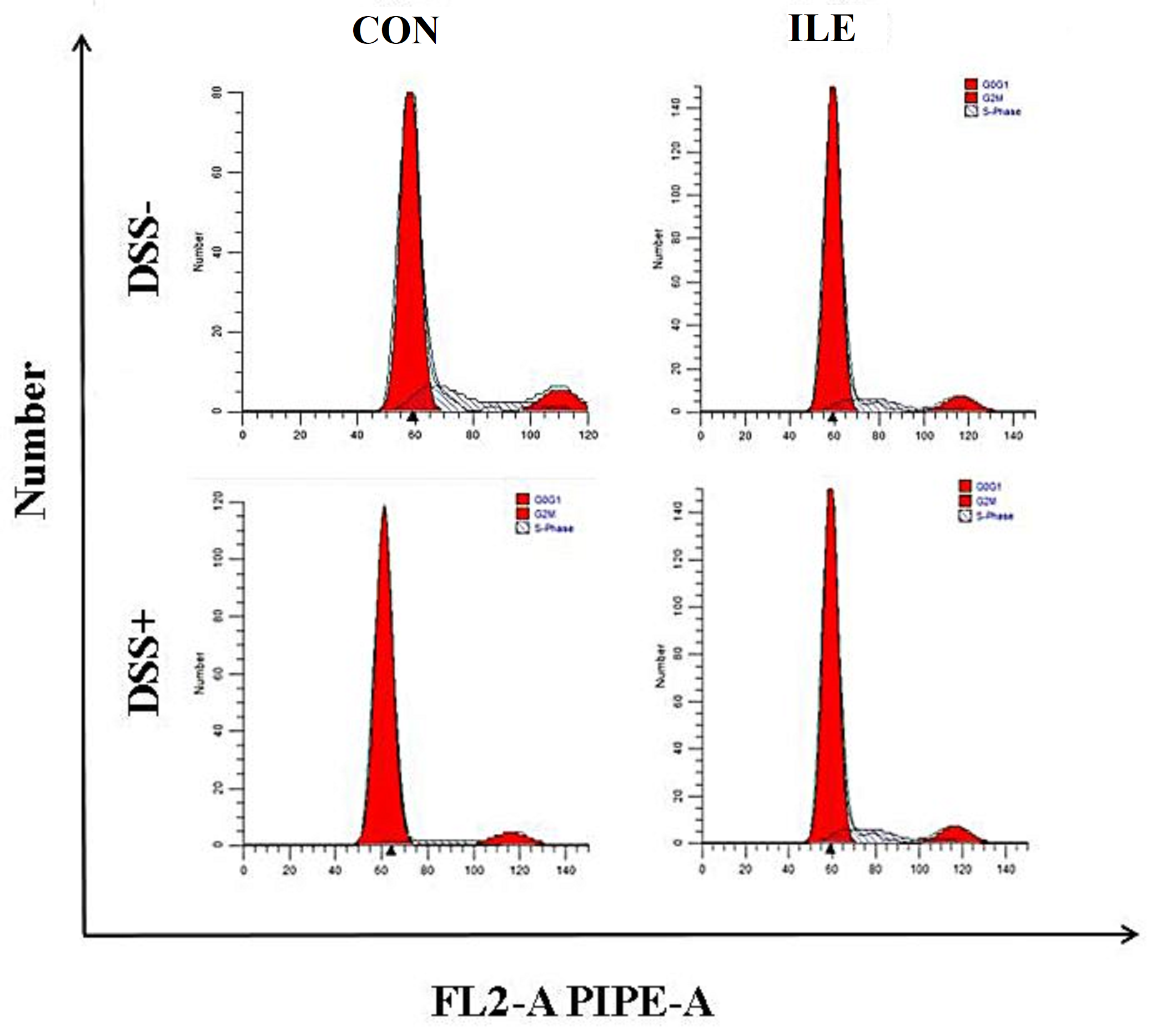

Supplement: Supplementary Figure 2 — DNA histogram of the cell cycle in the colonic epithelium cells of normal and DSS-challenge rats. The DNA histogram shows the colonic epithelium cell cycle in the G0/G1 phase (first peak), the G2 + M phase (second peak), and the S phase (region between the first and second peak). DSS -, drinking the ultrapure water; DSS +, drinking the ultrapure water with DSS; CON, l-alanine-supplemented diet; ILE, l-isoleucine-supplemented diet. [file Image_2.tif]

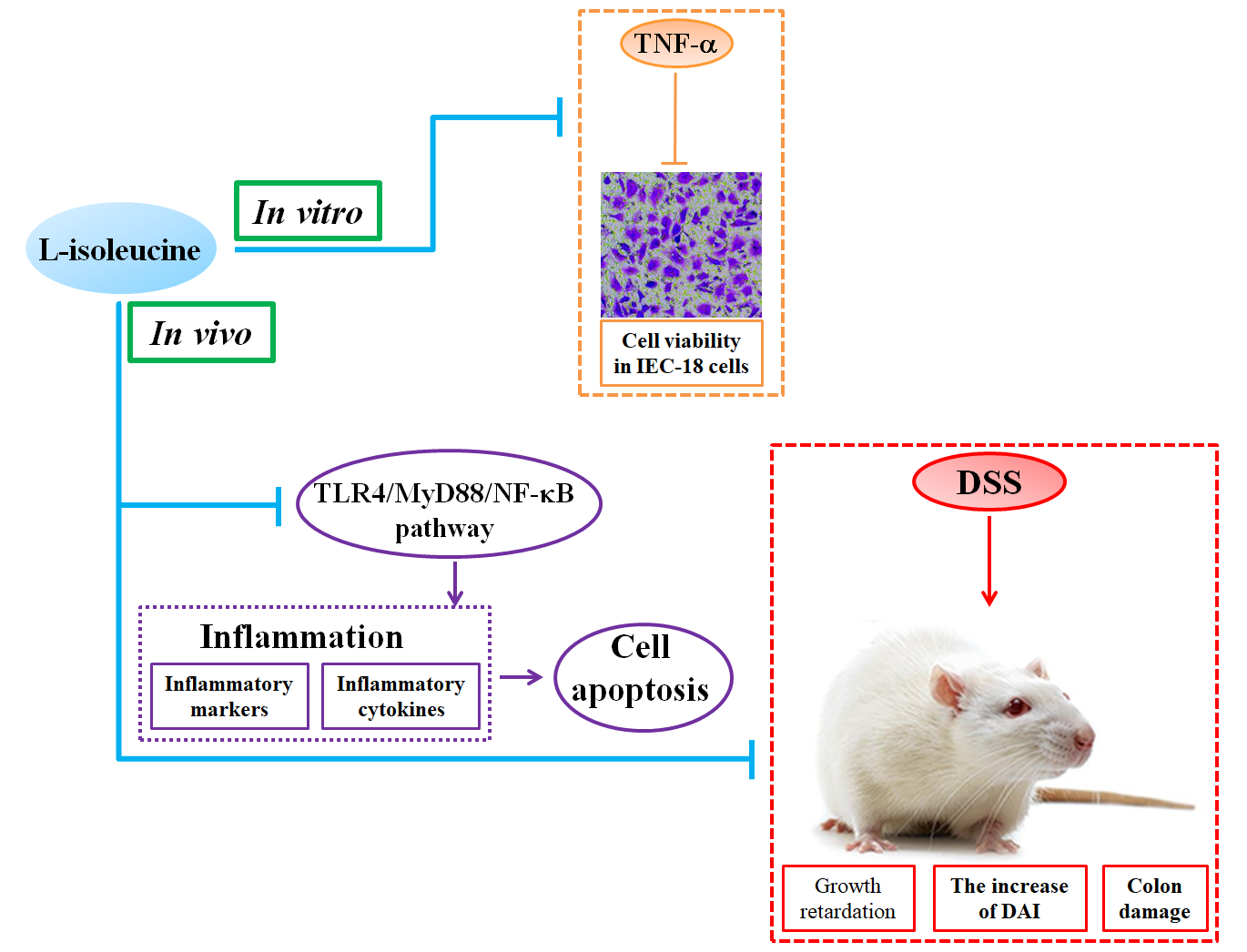

Supplement: Supplementary Figure 3 — Graphic abstract. [file Image_3.png]
