# Supplementary material for: l-Isoleucine Administration Alleviates DSS-Induced Colitis by Regulating TLR4/MyD88/NF-κB Pathway in Rats
Source: Front Immunol. 2022 Jan 11;12:817583. doi: 10.3389/fimmu.2021.817583 (PMC8787224; doi:10.3389/fimmu.2021.817583)
Supplement: Supplementary file 4 [file Table_1.docx]

Supplemental Table 1 Criteria for scoring disease activity index

| Score | Weight loss (%) | Stool consistency | Occult blood or gross bleeding |
| --- | --- | --- | --- |
| 0 | None | Normal stool | Negative |
| 1 | 1-5 | Loose stool | Negative |
| 2 | 5-10 | Loose stool | Hemoccult positive |
| 3 | 10-15 | Diarrhea | Hemoccult positive |
| 4 | > 15 | Diarrhea | Gross bleeding |

Disease activity index = (combined score of weight loss, stool consistency, and bleeding)/3

Stool consistency: Normal stool = well formed pellets; Loose stool = pasty stool that does not stick to the anus; Diarrhea = liquid stools that sticks to the anus.

Supplemental Table 2 Primers of genes

| Genes | Primer sequences (5’-3’) |
| --- | --- |
| *ZO-1* | F: ATGACCGAGTCGCAATGGTT |
|  | R: TCTATCCCTTGCCCAGCTCT |
| *MUC2* | F: CACCTACCTTGTTGTGGAGGC |
|  | R: ACAGGAGATGACATTGAGCTGG |
| *Claudin-1* | F: CCTCTTACCCAACACCACGG |
|  | R: GTGGCAAGCAGCAGTTCAAA |
| *TLR-4* | F: CATTGCTGCCAACATCATCCA |
|  | R: CCAGAGCGGCTACTCAGAAACT |
| *MyD88* | F: TTGCTAGCCTTGTTAGACCGT |
|  | R: CTCCTGTTTCTGCTGGTTGCG |
| *NF-κB* | F: TTTGATAACCGTGCCCCCAA |
|  | R: GTCAGCGTATGGGGGAGTTC |
| *β-actin* | F: GGAGATTACTGCCCTGGCTCCTAGC |
|  | R: GGCCGGACTCATCGTACTCCTGCTT |

ZO-1, zonula occluden 1; MUC2, mucin 2; TLR4, toll-like receptor 4; MyD88, myeloid differentiation primary response gene 88; NF-κB, nuclear factor-kappa B.
